# Morele dilemma’s rond gelijke kansen op gezondheid

**DOI:** 10.1007/s12508-022-00370-x

**Published:** 2022-11-03

**Authors:** Beatrijs Haverkamp, Marcel Verweij

**Affiliations:** Philosophy Group, Wageningen University, Wageningen, Nederland

**Keywords:** gemeentelijk volksgezondheidsbeleid, kansengelijkheid, gezondheidsverschillen, Local policy making, Equity, Health inequalities

## Abstract

In het kader van kansengelijkheid is het verbeteren van de gezondheid van achtergestelde groepen voor gemeenten een belangrijke maar lastige opdracht. Naast gebrek aan kennis over wat effectieve interventies zijn, kan er ook onzekerheid zijn over de vraag wat *moreel* gezien eigenlijk het beste is om te doen. Om een beeld te krijgen van deze ‘morele obstakels’ inventariseerden we bij de afdeling Volksgezondheid van de gemeente Utrecht welke ethische dilemma’s medewerkers tegenkomen in hun streven naar gelijke kansen op gezondheid. Op basis van vier dilemmabesprekingen met gemeentemedewerkers en filosofische literatuur over rechtvaardigheid, formuleren we hier vijf inzichten over wat het concreet betekent om in de gemeentelijke volksgezondheidspraktijk gelijke kansen op gezondheid te bevorderen. Te weten 1) kansengelijkheid bevorderen vereist dat medewerkers in de beleidspraktijk morele afwegingen maken; 2) kansengelijkheid vraagt om balanceren tussen idealisme en realisme; 3) feitelijke kansengelijkheid vereist meer dan formele kansengelijkheid; 4) kansengelijkheid kunnen we het beste bevorderen door prioriteit te geven aan degenen die het slechtst af zijn; 5) kansengelijkheid bevorderen vraagt om paternalisme. Deze inzichten, samen met de gebruikte gespreksstructuur, kunnen gemeentemedewerkers ondersteunen bij het maken van keuzen die bijdragen aan gelijke kansen op gezondheid.

**Digitaal aanvullende content:**

De online versie van dit artikel (10.1007/s12508-022-00370-x) bevat aanvullend materiaal, toegankelijk voor daartoe geautoriseerde gebruikers.

## Abstract (en)

From the perspective of social justice and equality of opportunity, it is an important yet difficult aim of local governments to enhance the opportunity to be healthy among disadvantaged groups. Apart from a lack of knowledge about effective interventions, there can be uncertainty about what is *morally* the best thing to do. To get a better understanding of what these moral challenges are, we made an inventory of the ethical dilemmas faced by employees of the public health department of the Dutch City of Utrecht in their aspiration to achieve health equity. Based on both four structured discussions of these dilemmas with municipality employees, and central ideas and distinctions from the philosophical literature on social justice, we formulate five insights on what it means to take equality of opportunity as ideal in local public health policy. That is, 1) fostering equal opportunities to be healthy demands that moral choices are made within policy practice and the execution of policies; 2) fostering equal opportunities to be healthy demands a balancing act between realism and idealism; 3) substantial equality of opportunity in health demands more than formal opportunity; 4) fostering equal opportunities to be healthy works best by giving priority to the worst-off; 5) fostering equal opportunities to be healthy requires paternalism. These insights, together with the structuring questions, can support local policymakers in public health in the decisions they need to make, by clarifying which considerations contribute to health equity.

## Inleiding

In een stad als Utrecht voelde 91% van de mensen met een hbo- of wo-opleiding zich in 2020 gezond, tegenover 57% van de mensen met alleen basisonderwijs of een mbo-opleiding [1]. De afgelopen jaren zijn sociale gezondheidsverschillen herhaaldelijk op de kaart gezet door adviesraden en maatschappelijke organisaties als een onrechtvaardige, complexe en hardnekkige ongelijkheid [2–4]. De coronacrisis heeft de urgentie van deze boodschap verder versterkt: de maatregelen tegen de virusverspreiding troffen de meest achtergestelde groepen het hardst en het is aannemelijk dat ze geleid hebben tot een vergroting van bestaande gezondheidsverschillen [5–7].

Beleid gericht op het verkleinen van gezondheidsverschillen is moreel gemotiveerd vanuit een algemeen ideaal van sociale rechtvaardigheid of kansengelijkheid. Voor ons onderzoek bij de gemeente Utrecht sluiten we aan bij de terminologie van kansengelijkheid omdat dit een van de drie uitgangspunten is van de Utrechtse gezondheidsnota ‘Gezondheid voor iedereen’ [8]. ‘Kansengelijkheid’ is een breed begrip uit de liberale politieke filosofie, een traditie die individuele vrijheid als een van de belangrijkste waarden ziet. Daarbij kent deze traditie een veelheid aan definities van vrijheid, en daarmee van kansengelijkheid, waaraan we in dit artikel geen recht kunnen doen. We focussen hier op ‘kansen op gezondheid’ die – zoals gezondheidsverschillen laten zien – sterk worden beïnvloed door sociaaleconomische positie, zoals bepaald door opleiding en inkomen. Kansen op gezondheid zijn dus op een complexe manier verweven met andere vormen van kansen(on)gelijkheid.

Het verbeteren van de gezondheid van benadeelde groepen is voor veel gemeenten dan ook een belangrijke, maar lastige opgave. Vaak zijn de obstakels groot en is de praktijk weerbarstig, bijvoorbeeld omdat de oorzaken van gezondheidsproblemen samenhangen met armoede, milieuvervuiling, discriminatie of de uitwassen van een vrijemarkteconomie. Ook kan gebrek aan kennis van wat effectieve interventies zijn – en wat niet – een verkleining van de gezondheidskloof bemoeilijken. Maar soms bestaan er ook ‘morele obstakels’: onzekerheid over de vraag wat *moreel* gezien eigenlijk het beste is om te doen.

In de ethische literatuur over gezondheidsverschillen vinden we ten minste drie kwesties waarover discussie bestaat. Ten eerste de vraag welke gezondheidsverschillen onrechtvaardig zijn en welke niet, of minder. Anders gezegd: welke groepen met een gezondheidsachterstand verdienen prioriteit bij gezondheidsbevorderende interventies? In de afwegingen over het antwoord op die vraag spelen de aard van de oorzaken en van de gevolgen van gezondheidsachterstanden een rol, en ook de ernst ervan [9]. Een tweede kwestie is de vraag in hoeverre een overheid zich mag bemoeien met de gezondheid van individuele burgers. Anders dan in discussies in de medische ethiek, speelt *informed consent *in de ethiek van volksgezondheid niet zo’n centrale rol. Dat is begrijpelijk omdat een overheid doorgaans niet op individueel, maar op groepsniveau intervenieert. Enige mate van paternalisme is daarmee onvermijdelijk en in de volksgezondheidsethiek breed geaccepteerd [10]. Een derde punt van discussie is de vraag in welke mate verschillende politieke, maatschappelijke en commerciële actoren verantwoordelijk zijn voor de verkleining van gezondheidsachterstanden. Verschillende overwegingen spelen hier een rol, zoals de mate waarin een actor heeft bijgedragen aan gezondheidsproblemen, de mate waarin een actor in de positie is gezondheidsproblemen te voorkomen of te verminderen, en wat de (wettelijk of maatschappelijk bepaalde) rol en taak zijn met betrekking tot de publieke gezondheid [11, 12].

Om een beeld te krijgen van wat de concrete morele obstakels in de lokale beleidspraktijk zijn, inventariseerden we bij de afdeling Volksgezondheid van de gemeente Utrecht welke ethische dilemma’s medewerkers tegenkomen in hun streven naar gelijke kansen op gezondheid. Morele onzekerheid laat zich door haar aard niet beantwoorden door (alleen) meer feitenkennis, maar vraagt om reflectie op waarden en principes. Om die reden organiseerden we dilemmabesprekingen met uitdrukkelijke aandacht voor kansengelijkheid als een te bevorderen waarde. Wat leren deze casusbesprekingen ons over de betekenis van het abstracte ideaal van kansengelijkheid voor de lokale volksgezondheidsbeleidspraktijk? In deze bijdrage bespreken we vijf inzichten die we in de discussies hebben opgedaan, en we onderbouwen deze inzichten door ze te relateren aan recente literatuur over rechtvaardigheid en gezondheid.

## Methode

De eerste auteur maakte een inventarisatie van ethische dilemma’s door tussen september 2019 en medio maart 2020 gesprekken te voeren met medewerkers van de afdeling Volksgezondheid (VG) van de gemeente Utrecht. Deze gesprekken vonden hoofdzakelijk plaats op basis van ‘aanmelding’: medewerkers werden op de hoogte gebracht van het onderzoek door een presentatie en een e‑mail waarin ze werden uitgenodigd zich te melden wanneer ze in hun dagelijkse werk met dilemma’s te maken hadden waarbij gelijke kansen op gezondheid een rol speelden. De individuele gesprekken hierover waren gericht op het verhelderen van het dilemma en de precieze situatie waarin dit zich voordeed. Deze gesprekken betroffen geen formele of gestructureerde interviews, maar werden gevoerd aan de hand van vragen over de achtergrond en motivaties van beleidskeuzen die tot het dilemma leidden, over de gevolgen van handelingsopties en over eventuele alternatieven. Na ongeveer 25 medewerkers van de verschillende teams bij VG te hebben gesproken (Beleid en onderzoek, Leefomgeving, Co-Creatie & Wijken, Culturele aspecten, Dak- en thuislozenzorg, Team Volwassenen) gingen we met een klankbordgroep na welke thema’s nog ontbraken. Op advies van de klankbordgroep – bestaande uit twee medewerkers van VG Utrecht, één van Pharos, één onderzoeker van het RIVM en één epidemioloog van het UMC Amsterdam – deed de eerste auteur nog specifiek navraag bij het team Epidemiologie en het team Gezond opgroeien om te horen of zich daar dilemma’s voordoen, en zo ja welke. Dit resulteerde in een lijst van dilemma’s in de volle breedte van het volksgezondheidsbeleid – van sturing op gezond gedrag en onderzoek naar gezondheidsniveaus tot beleid ter verbetering van de leefomgeving.

Vervolgens organiseerden we in januari–februari 2021 vier online casusbesprekingen, waarvoor medewerkers zichzelf konden aanmelden (maximaal vijf per bespreking). Voor drie besprekingen selecteerden we dilemma’s uit de lijst, om de diversiteit aan dilemma’s te dekken. Deze werden uitgeschreven en voorgelegd aan de medewerker die het dilemma eerder had aangedragen om te controleren of de beschrijving feitelijk juist en de morele vraag herkenbaar was (zie de bijlage in de digitaal aanvullende content). Omdat de besprekingen tijdens de COVID-19-pandemie plaatsvonden, formuleerden we als onderzoekers – in samenspraak met een gemeentemedewerker die medeverantwoordelijk was voor het coronabeleid – ook een vierde dilemma over immuniteitsbewijzen, waarvan de verwachting toen was dat deze vraag zich op korte termijn zou voordoen. Zie het kader voor een korte beschrijving van de vier casussen en de bijlage (in de digitaal aanvullende content) voor de volledige beschrijvingen.

Ter structurering van de casusbespreking formuleerden we een aantal vragen die kansengelijkheid en rechtvaardigheid centraal stelden:

- In welke zin is hier sprake van kansenongelijkheid?
- Waarom is deze ongelijkheid problematisch, of zelfs onrechtvaardig?
- Wat voor zorgplicht heeft de gemeente?
- Welke opties zijn er om de ongelijkheid (effectief) te beperken?
- Welke optie heeft op grond van de bespreking de voorkeur?

Deelnemers kregen de casus en de structurerende vragen vooraf te lezen. Tijdens de discussie reflecteerden deelnemers per vraag op de casus en op elkaars overwegingen. Onze rol als onderzoekers was hierbij primair faciliterend van aard: de tweede auteur leidde de discussie aan de hand van bovenstaande vragen, de eerste auteur vatte na afloop de hoofdpunten samen.

Op basis van een opname van het gesprek maakten we korte gespreksverslagen. Deze hebben we gebruikt om tot vijf inzichten te komen. De inzichten zijn *geen analyse *van de casusbesprekingen, maar het resultaat van *onze reflectie op overwegingen* die in de besprekingen naar voren werden gebracht en die wij relateerden aan filosofische rechtvaardigheidstheorieën.

### Korte beschrijving van de casussen

(Zie de digitaal aanvullende content voor volledige casusbeschrijvingen.)

1. Om gelijke kansen op gezond eten te bevorderen, stimuleert de gemeente de ontwikkeling van een gezonde voedselomgeving. In wijken waar de minst gezonde mensen wonen, is het – om verschillende redenen – moeilijker voor aanbieders van gezond voedsel om zich te vestigen dan in de wijken waar de nood aan gezonde voeding minder hoog is, maar waar de aftrek ervan groter zal zijn. Hoe stel je dan prioriteiten in het stimuleren van een gezonde voedselomgeving?
2. Voor mensen bij wie sociale problemen zich opstapelen schieten algemene publieke voorzieningen al gauw tekort. Mensen belanden soms in zulke uitzichtloze situaties dat het aankomt op noodinterventies ten behoeve van hun gezondheid. Hoe ver ga je daarin als gemeentemedewerker, en wanneer leg je je bij een situatie neer?
3. Gelijke kansen op gezond opgroeien stimuleren de gemeente door collectieve opvoedondersteuning aan te bieden. Hoogopgeleide ouders weten de adviseurs opvoedondersteuning sneller te vinden dan ouders in sociaaleconomisch kwetsbaardere posities, terwijl eerstgenoemden wellicht ook op eigen kracht oplossingen zouden vinden. Wat is dan goed om te doen: de vragende, hoogopgeleide ouders blijven ondersteunen, of meer investeren in het bereiken van kwetsbaardere ouders die zich niet uit eigen beweging melden?
4. Dankzij de steeds grotere beschikbaarheid van sneltests en vaccinaties wordt het mogelijk om allerlei bedrijven, (culturele) instellingen en (sport)clubs te heropenen, maar dan alleen voor burgers met een recent test- of vaccinatiebewijs. Wat voor instanties moeten wel of niet met een immuniteitsbewijs toegankelijk worden? En onder welke voorwaarden?

## Resultaten

Een algemeen resultaat van deze besprekingen was dat de discussies voor de direct bij de casus betrokken medewerkers een verdiepende reflectie boden op een probleem uit de dagelijkse praktijk. De deelnemers vonden de besprekingen waardevol omdat ze zich konden bezinnen op de keuzen die in VG-werk gemaakt worden, en ook op hun meer fundamentele motivatie in het werk. Sommigen gaven aan daarbij niet zozeer de oplossing van het dilemma van belang te vinden, als wel een reflectie op wat eigenlijk nagestreefd wordt in beleid met kansengelijkheid als uitgangspunt. Wat houdt dat ideaal eigenlijk in? Terugkijkend op de besprekingen komen we tot de volgende vijf inzichten.

### Kansengelijkheid bevorderen vereist dat medewerkers in de beleidspraktijk morele afwegingen maken

We beginnen met de constatering – én aanname van ons onderzoek – dat werken aan kansengelijkheid betekent dat er in de beleidspraktijk morele afwegingen moeten worden gemaakt die voorbijgaan aan politieke keuzen. Zowel de specifieke uitwerking als de uitvoering van beleid vraagt om specifieke morele kwaliteiten van medewerkers die in de beleidspraktijk werken [13]. Vooral bij de bespreking van de vraag hoe je rechtvaardig omgaat met inwoners met geclusterde sociale problemen (casus 2) werd dit duidelijk. Bij VG behandelt een klein team de zorgvragen van burgers voor wie standaard publieke woon- en zorgvoorzieningen niet voldoen. Het bevorderen van kansengelijkheid betekent dan vaak: specifieke mensen *ongelijk* behandelen – bijvoorbeeld door hen meer zorg en hulp te bieden bij het vinden van woonruimte dan anderen. Verantwoorde beslissingen vergen hier in de eerste plaats een goed oordeelsvermogen: medewerkers moeten inschatten wat moreel relevante verschillen zijn tussen individuele hulpvragers, om te beoordelen wanneer het geoorloofd en/of een plicht is om extra hulp te bieden. Het vraagt vervolgens om bescheidenheid over het eigen oordeelsvermogen om dit oordeel bij twijfel voor te leggen aan collega’s. Wanneer de uiteindelijke conclusie luidt dat een uitzonderingsbehandeling inderdaad gerechtvaardigd is, is er moed nodig van de betrokken medewerker(s) om naar dit oordeel te handelen en een uitzondering op de algemene richtlijnen te maken. Ook kan het moed vragen om breder spelende problemen aan te kaarten. Bij het werken aan een gezonde voedselomgeving (casus 1) zien we het belang van nog een andere kwaliteit: het vermogen om kleine verbeteringen te blijven waarderen (bijvoorbeeld moestuininitiatieven door bewoners die waarschijnlijk toch al gezond eten) en ondertussen langetermijnidealen voor ogen te houden (een gezond voedselaanbod in de hele stad). Dat is direct gerelateerd aan een tweede inzicht.

### Kansengelijkheid bevorderen vraagt om het vinden van een balans tussen idealisme en realisme

Bij iedere bespreking werd al snel duidelijk dat échte kansengelijkheid een onhaalbaar ideaal is. De casus over het stellen van prioriteiten bij het werken aan een gezonde voedselomgeving (casus 1) vormt daarvan een goede illustratie. In een (relatief) vrije markt wordt de stedelijke voedselomgeving in belangrijke mate bepaald door factoren waarop lokale overheden doorgaans weinig invloed hebben en die een gelijk en gezond voedselaanbod in de weg staan. Voorbeelden zijn de prijsstellingen van voedsel en de vestigingsvrijheid van ondernemers, en ook de werking van sociale normen over wat ‘goed’ of ‘normaal’ eten is. Hierdoor blijkt het vaak moeilijker om het aanbod van gezond voedsel te vergroten in wijken waar de gemiddelde gezondheid slechter is, dan in meer welvarende en gemiddeld gezondere wijken.

In filosofische discussies over rechtvaardigheid is dit probleem geadresseerd met de vraag hoe je dichter bij perfecte rechtvaardigheid komt in een ‘niet-ideale’ wereld. Amartya Sen heeft in een discussie met John Rawls laten zien dat het *ideaal* van sociale rechtvaardigheid als zodanig nog niet vertelt hoe we moeten handelen in een wereld die helemaal niet aan dat ideaal voldoet [14]. Is het bijvoorbeeld beter om een grote groep die slechts een beetje van het ideaal af leeft er effectief dichter bij te brengen, of is het beter te focussen op de kleinere groep voor wie het ideaal ver weg, zo niet onhaalbaar is? En is het beter te proberen om structurele en systemische oorzaken te veranderen, of om in te zetten op verbeteringen binnen het systeem?

Bij het streven naar kansengelijkheid in het voedselaanbod bewegen VG-medewerkers constant heen en weer tussen idealisme en realiteit. Enerzijds kaarten ze de structurele oorzaken van ongelijkheid aan – door te lobbyen voor regulering van het voedselaanbod bij de nationale politiek, door ondernemers en organisaties in de stad aan te spreken op sociale verantwoordelijkheid, door in gesprek te gaan met andere gemeentelijke afdelingen, enzovoort. Anderzijds zetten ze in op de best mogelijke uitkomsten *binnen* de gegeven en verre van optimale structuren – door gebruik te maken van de bevoegdheden met betrekking tot vestigingsvrijheden van cateraars, door initiatieven voor gezond eten in wijken en buurten te stimuleren, en door in eigen huis voor een gezond voedselaanbod te zorgen. Daarmee laten medewerkers zien dat ze werken aan wat we hieronder ‘feitelijke kansengelijkheid’ noemen.

### Feitelijke kansengelijkheid vereist meer dan formele kansengelijkheid

Een specifieke uitwerking van de spanning tussen realisme en idealisme zien we in het onderscheid tussen formele en feitelijke kansengelijkheid. Formele kansengelijkheid betreft het idee dat een formeel gelijke toekenning van rechten resulteert in gelijke kansen. Het idee van feitelijke kansengelijkheid erkent echter dat dit in werkelijkheid niet zo uitpakt: échte (feitelijke) kansengelijkheid vraagt om ongelijke behandeling om gelijke mogelijkheden te realiseren [15]. In dit licht is de zogenaamde *capabilities*-benadering van Amartya Sen en Martha Nussbaum relevant [16]. Capabilities zijn voor Sen en Nussbaum de kansen die mensen feitelijk hebben: hun werkelijke, praktische mogelijkheden in het leven. Daarbij gaat het om de wisselwerking tussen materiële, fysieke en sociale omstandigheden, en de biologische en psychische eigenschappen van het individu. Feitelijke kansengelijkheid vraagt om een goede combinatie van contextuele en individuele factoren.

Het verschil tussen formele en feitelijke kansen werd onder meer duidelijk in de discussie over de (toen nog hypothetische) toepassing van COVID-19-immuniteitsbewijzen (casus 4). De toegankelijkheid van vaccinaties – waarmee de kansenongelijkheid in deze casus al begint – is formeel voor iedereen gelijk: het stond iedere Utrechter vrij om zich te laten vaccineren (volgens een landelijk bepaalde volgorde). Maar in werkelijkheid bestaan er niet te onderschatten ongelijkheden door zowel materiële als sociaal-culturele drempels: de fysieke afstand tussen huis en vaccinatielocatie verschilt, en de beschikbaarheid van eigen vervoer en eventuele ov-kosten creëren verschillen (voor wie weinig te besteden heeft is een busritje al een relatief grote uitgave). Daarnaast zijn er ongelijkheden in de mate waarin mensen zich goed kunnen informeren over de voor- en nadelen van vaccineren. Zeker in het geval van laaggeletterdheid maakt de tegenstrijdige informatie via onder andere sociale media het mensen lastig om een weloverwogen beslissing te nemen.

Formele kansengelijkheid is zo bezien een prima idee voor een ideale wereld, maar in de werkelijke wereld kunnen we hier geen genoegen mee nemen. Het bevorderen van feitelijke kansengelijkheid is echter veeleisend en kan ook moreel gezien lastig zijn. Dat brengt ons bij een vierde inzicht.

### Kansengelijkheid kunnen we het beste bevorderen door prioriteit te geven aan degenen die het slechtst af zijn

Strikte feitelijke kansengelijkheid realiseren is niet alleen een onhaalbaar, maar volgens sommigen ook een onwenselijk doel – er zullen altijd verschillen bestaan tussen mensen en daarmee tussen de feitelijke mogelijkheden die mensen hebben. Al die verschillen wegnemen zou simpelweg te veel overheidsingrijpen vergen. Eén veelbesproken argument tegen een focus op louter meer gelijkheid, is het zogenaamde *levelling down*-argument: sommige verschillen in gezondheid kunnen we alleen verkleinen door gezondere mensen ziek te maken – wat natuurlijk moreel onaanvaardbaar zou zijn [17].

Het garanderen van minimumniveaus is dan mogelijk een realistischer en aanvaardbaarder ideaal. Dat gaat ervan uit dat het moreel gezien primair van belang is dat mensen *voldoende* hebben of kunnen, in plaats van dat mensen *hetzelfde* hebben of kunnen [18]. Het waarborgen van een welzijns- of welvaartsminimum hoeft daarbij geen laag niveau te betekenen: we kunnen de drempelwaarde zo hoog of laag maken als ons goeddunkt. Maar hoeveel dan genoeg is, is politiek én ethisch lastig vast te stellen [19]. Wanneer is een gezinssituatie ‘gezond’ genoeg om zonder ondersteuning te kunnen (casus 3)? Of hoeveel gezonde voedselaanbieders moet een buurt hebben (casus 1)?

Een iets andere benadering – onder andere verwoord door politiek filosofen Jonathan Wolff en Avner De-Shalitt – stelt daarom dat we ons moeten richten op wie het slechtst af zijn. Die groepen mensen moeten prioriteit krijgen in de verdeling van aandacht en middelen. Wie heeft de *minste* kansen op een gezond leven gegeven allerlei (elkaar versterkende) maatschappelijke risicofactoren? [20]. Vanuit dat perspectief ligt het voor de hand om in het aanbieden van collectieve opvoedondersteuning als gemeente meer tijd en moeite te steken in ondersteuning van gezinnen in achterstandssituaties, dan van gezinnen die het relatief goed hebben. En hetzelfde geldt voor investeringen in een gezonde voedselomgeving: kansengelijkheid kunnen we het best concreet maken voor de beleidspraktijk door keuzen te maken waarin prioriteit wordt gegeven aan gezondheidsbevordering en -bescherming in ‘achterstandswijken’.

### Kansengelijkheid bevorderen vraagt om paternalisme

Het bevorderen van kansengelijkheid – of dat nu via minimumniveaus of prioriteiten voor de minst bevoordeelden gebeurt – vraagt om ‘doelgroepenbeleid’. Dat brengt een risico van betutteling en stigmatisering met zich mee [21]. Het perspectief van ‘proportioneel universalisme’ benadrukt een generiek beleid, en is daarom door onder andere de WRR voorgesteld als een meer inclusieve benadering [2]. Maar wanneer generiek beleid proportioneel naar gezondheidsachterstand wordt uitgevoerd, valt in de praktijk niet te ontkomen aan doelgroepenbeleid. Op specifieke groepen gericht beleid gaat gepaard met een oordeel over wat die groep mensen nodig heeft. Een oordeel dat inherent iets paternalistisch heeft, omdat het wordt gevormd vanuit de veronderstelling dat je als gemeente weet wat goed is voor je inwoners – ondersteuning bij het opvoeden van kinderen bijvoorbeeld (casus 3).

De bekendste problematisering van paternalisme is beschreven door de liberaal John Stuart Mill. Hij stelde dat er in principe maar één legitieme reden is om iemands handelen of gedrag te beïnvloeden: namelijk wanneer dit handelen anderen schaadt. Alleen in het geval van kinderen en ‘incompetente’ volwassenen is het gerechtvaardigd om hun vrijheid te beperken ten behoeve van hun eigen welzijn [22]. Met deze liberale bril zien we echter ook dat paternalisme in het streven naar *kansen*gelijkheid niet problematisch is: wanneer gelijkheid in *kansen* het doel is, laat je mensen feitelijk vrij om die kansen wel of niet te benutten. Bij het aanbieden van collectieve opvoedondersteuning zien medewerkers reden om sommige ouders meer (of andere) ondersteuning te bieden – sommige kinderen lopen door een sociale omgeving nu eenmaal meer risico om het ‘verkeerde pad op te gaan’ of om met huiselijk geweld in aanraking te komen. Maar de ouders die extra ondersteuning in de opvoeding krijgen aangeboden *hoeven* hier geen gebruik van te maken.

Ook meer in het algemeen is een paternalistisch beleid niet problematisch zolang gewaarborgd wordt dat individuen respectvol en als gelijkwaardig worden bejegend [10]. Dat is des te belangrijker als specifieke kwetsbare groepen of individuen worden aangesproken – de beste bedoelingen kunnen immers als kleinerend of stigmatiserend, en daarmee als respectloos, worden ervaren [23]. Tegelijk mogen we niet over het hoofd zien dat in een samenleving waarin ‘zelfredzaamheid’ breed wordt gepropageerd, het vragen van hulp moeilijk kan zijn [24]. Het aanbod om extra ondersteuning te krijgen kan dan juist ook als bevrijdend worden ervaren in plaats van als respectloos.

## Beschouwing en conclusie

Voordat we ingaan op de vraag hoe de hier besproken inzichten kunnen helpen om kansengelijkheid te bevorderen, vatten we ze nog eens samen. De inzichten verschillen immers van elkaar, terwijl ze tegelijk nauw met elkaar samenhangen. Zo is het balanceren tussen idealisme en realisme gerelateerd aan het onderscheid tussen formele en feitelijke kansengelijkheid. Formele kansengelijkheid valt gemakkelijk te realiseren, terwijl het ideaal van feitelijke kansengelijkheid moeilijk en nooit volledig haalbaar is. De benadering van dat ideaal behoeft dan wel weer een flinke dosis realisme: werken aan feitelijke kansen vraagt om oog voor de realiteit waarin mensen leven. Prioriteit geven aan degenen die het slechtst af zijn, is daarbij een realistische en moreel verdedigbare invulling van het ideaal van kansengelijkheid. Streven naar ‘genoeg voor iedereen’ *kan* er ook één zijn, maar minimumniveaus vaststellen zal moreel gezien altijd iets willekeurigs hebben. Zowel denken in termen van minimumniveaus als denken in termen van prioriteit voor de minst bevoordeelden resulteert in doelgroepenbeleid. Daarbij is paternalisme een onvermijdelijke en niet per se problematische bijkomstigheid.

Bij het eerste inzicht hebben we geconcludeerd dat werken aan kansengelijkheid veel vraagt van degenen die in de beleidspraktijk werken. We noemden daarbij het belang van specifieke kwaliteiten, zoals een moreel oordeelsvermogen, nederigheid en moed, en een gezonde mix van realisme en idealisme. Zonder het belang van heldere regels of principes te ondermijnen, willen we benadrukken dat de toepassing van regels altijd ook om een interpretatie van de regels vraagt. Die interpretatie is geen objectief of rationeel oordeel, maar doet ook een beroep op de morele intuïties, gevoelens en motieven van gemeentemedewerkers. De gespreksstructuur zoals beschreven in de methodesectie helpt om deze intuïties en motieven te expliciteren, en zo bewuster en beter te verantwoorden keuzen te maken die kansengelijkheid bevorderen. De overige vier inzichten bieden daarbij elk specifieke aandachtspunten: het formuleren van realistische ambities, zonder langetermijnidealen op te geven (het tweede inzicht); oog hebben voor de wisselwerking tussen materiële, fysieke en sociale omstandigheden, en biologische en psychische factoren (het derde inzicht); een bewuste keuze maken voor het prioriteit geven aan de meest achtergestelde groepen (het vierde inzicht); erkennen dat werken aan kansengelijkheid altijd iets paternalistisch heeft, en daarbij zorg dragen voor een respectvolle benadering van mensen als gelijkwaardige burgers (het vijfde inzicht).

Kansengelijkheid is een essentiële waarde in het lokale gezondheidsbeleid en ethische reflectie is cruciaal om dat beleid op een heldere en verantwoorde manier in te vullen.
