# Supplementary material for: Moral dilemmas around health equity
Source: TSG. 2022 Nov 3;100(4):156–62. [Article in Dutch] doi: 10.1007/s12508-022-00370-x (PMC9633034; doi:10.1007/s12508-022-00370-x)
Supplement: Supplementary file 1 [file 12508_2022_370_MOESM1_ESM.docx]

**Bijlage – Vier casussen**

*Casus 1: Gezonde voedselomgeving*

De gemeente Utrecht heeft zich met haar nota Volksgezondheid gecommitteerd aan het stimuleren van een gezonde voedselomgeving. Waar de gemeente nu nog grotendeels afhankelijk is van wat partijen in de stad aan initiatieven opzetten, bestaat er een kans dat gemeenten op termijn meer bevoegdheden zullen krijgen om effectiever te sturen in de voedselomgeving. Dat maakt de vraag naar hoe prioriteiten te bepalen nog urgenter.
Toegang tot gezonde voeding lijkt een belangrijke factor om de hoge prevalentie van overgewicht in sommige wijken terug te brengen. In die wijken is de nood aan een gezonde voedselomgeving groter dan in andere. Het is echter niet zonder meer vanzelfsprekend om als eerste in te zetten op de wijken waar de nood het hoogst is: voor mensen die weinig te besteden en andere sociale problemen hebben, heeft gezond eten mogelijk en logischerwijs niet de hoogste prioriteit. Ook als mensen het belangrijk vinden, geldt dat gezonde voeding relatief duur is (gekeken naar prijs/calorieën). Dat maakt het voor ‘gezonde voeding’ aanbieders minder aantrekkelijk zich te vestigen in armere stadsdelen. Welke wijken dan voorrang te geven in het stimuleren van een gezonde voedselomgeving: die waar de gezondheidsnood het hoogst is of juist de meer welvarende buurten?

*Casus 2: Clustering sociale problemen*
Bij complexe problematiek lopen VG-medewerker gemakkelijk aan tegen de grenzen van wat sociale publieke voorzieningen te bieden hebben. Individuele situaties zijn soms zo uitzichtloos dat alleen heel specifieke noodinterventies nog kunnen helpen. Ter illustratie een casus zoals bekend bij Team Volwassenen:  
Een cliënt kampt sinds 20 jaar met ernstige GGZ-problematiek met o.a. agressief gedrag. Sinds enkele maanden woont hij bij zijn ouders omdat er op dit moment geen passende plek is. De zoektocht naar geschikte woonruimte is verder vertraagd omdat er eerst nog een uitspraak van de rechter moest komen en zittingen door Corona enorme vertraging hebben opgelopen. Terwijl ze wachten op een passende plek, nemen de problemen van de man toe met slapeloosheid en psychisch lijden voor zijn ouders als gevolg.
Vanuit Volksgezondheid wordt m.n. de gezondheid van cliënten en betrokken familie in het oog gehouden. Dit schuurt soms met het belang van bijvoorbeeld de afdeling Veiligheid waar het verminderen van overlast bovenaan staat, en met de beperkte mogelijkheden van overheid, sociale woonvoorzieningen en zorginstellingen. Dat de gezondheid van een cliënt niet altijd van het hoogste belang wordt geacht door alle partijen is veelal moeilijk uit te leggen aan de cliënt, familie en betrokken hulpverleners. Dit leidt niet alleen tot frustraties, maar ook tot dilemma’s bij VG-medewerkers: hoe hard moet je je best doen – door extra telefoontjes, overtuigingskracht e.d. – om de gezondheid en het welzijn van deze individuen wel centraal te stellen? En wanneer leg je je bij de situatie neer?

*Casus 3: Collectieve opvoedondersteuning*
Met de collectieve aanpak opvoedondersteuning zet VG in op preventief, tijdig en snel inspelen op veelvoorkomende vragen die leven in de wijk op het gebied van opvoeden en opgroeien. Uitgangspunt is om dit samen te doen met groepen ouders of jongeren. Het houdt concreet in dat er bijeenkomsten over verschillende onderwerpen worden georganiseerd voor ouders die vragen hebben over dezelfde thema’s.
De gemeente vervult hiermee haar zelf opgevatte taak om de sociale basis in wijken te versterken, door ouderschap te versterken en het mogelijk te maken dat ouders en jongeren elkaar ondersteunen bij opvoedvragen en zorgen die horen bij de gewone ontwikkeling van kinderen. Zo blijven kleine vragen klein en is de continuïteit van het aanbod in de wijk gewaarborgd.

Hoogopgeleide ouders weten de adviseurs collectieve opvoedondersteuning sneller te vinden dan ouders in sociaaleconomisch kwetsbaardere posities. Hoogopgeleide ouders hebben ook hun problemen, maar de vraag is of ze hier niet al op eigen kracht oplossingen voor zouden vinden. Een zorg die leeft bij team Gezond en Veilig Opgroeien is dan ook dat deze ouders de tijd innemen van ouders die het eigenlijk meer nodig hebben. Wat is goed om te doen: deze hoogopgeleide ouders blijven ondersteunen, of meer tijd en energie steken in het vinden van aansluiting bij ouders die zich niet uit eigen beweging melden?

*Casus 4: Covid19-immuniteitsbewijzen*

De kans bestaat dat er vanwege de Covid19 pandemie nog langere tijd (mogelijk tot in de herfst) veel preventie- en bestrijdingsmaatregelen in stand moeten blijven. Dankzij de steeds grotere beschikbaarheid van sneltesten en vaccinaties wordt het mogelijk om allerlei bedrijven, (culturele) instellingen en (sport)clubs te heropenen, maar dan alleen voor burgers met een recent test- of vaccinatiebewijs. Dit brengt allerlei dilemma’s met zich mee, maar het wordt gezien als een noodzakelijke stap om het maatschappelijk leven weer zo goed mogelijk te laten draaien, en ook om het welzijn van burgers te beschermen. De stad Utrecht mag mede sturing geven aan de implementatie en handhaving van dit beleid. Wat voor instanties moeten wel of niet met een immuniteitsbewijs toegankelijk worden? En onder wat voor voorwaarden?
